# The Earthquake at Charleston

**Published:** 1886-10-20

**Authors:** 


THE EARTHQUAKE AT CHARLESTON.—With all that has been said of the effects of this terrible calamity upon the citizens of Charleston, perhaps few have thought of the increased labors and responsibilities that the physicians of that afflicted city have incurred. Whilst themselves not exempt from the perils and sufferings of such a calamity, they are yet expected to encounter the increased professional emergencies that always exist under such appalling circumstances. The calls of humanity the physician cannot refuse ; and it is the glory of the profession that they have always been brave and true under the most trying circumstances of pestilence, war, and dreadful catastrophies so common in these times. We deeply feel for the members of the profession in Charleston, and think they ought to be especially remembered by those who are dispensing to the needs of the suffering, and that when Governmental appropriations are made for the relief of the people, as will doubtless be done, special compensation will be extended to those physicians who, amid the terrible trials and horrors resulting from the recent earthquake, have labored so faithfully for the relief of their suffering fellow-citizens.

